# Reversing the trend: interventions to treat intracranial haemorrhage associated with anticoagulation

**DOI:** 10.1007/s00415-016-8198-9

**Published:** 2016-06-20

**Authors:** D. McLauchlan, N. P. Robertson

**Affiliations:** Institute of Psychological Medicine and Clinical Neuroscience, Cardiff University, University Hospital of Wales, Heath Park, Cardiff, CF14 4XN UK

Spontaneous intracranial haemorrhage (ICH) is less common as a cause of stroke than ischaemia, but it has significantly worse morbidity and mortality. To some extent this reflects, the different demographics of the populations affected, but a lack of effective therapeutic options is also a contributory factor. Whilst ICH as a result of underlying vascular malformations or other structural lesions offers certain neuroradiological and/or neurosurgical possibilities to prevent recurrence, interventions to reverse damage caused by the index event remain limited regardless of aetiology. As a result, current management is mainly supportive and includes reversal of anticoagulation where appropriate, blood pressure control, prevention of hyperglycaemia and pyrexia, and the treatment of emergent complications, such as seizures.

Anticoagulant and antiplatelet medications are often associated with ICH and adversely affect outcome. Patients are treated with these agents for a variety of reasons, including primary or secondary prevention of cardiac and cerebral ischaemic events. Historically, there has been a choice been between aspirin (or other antiplatelet agents) or a coumarin. However, recently a number of new agents have been developed (novel oral anticoagulants—NOACs), and have potential advantages over Warfarin, including a lower risk of haemorrhagic complications for an equivalent level of thrombo-embolic risk reduction and the lack of requirement for international normalised ratio (INR) monitoring. However, none of the NOACs has a specific agent to reverse their action in the event of haemorrhagic complications.

This month, journal club focuses on reversal of drugs which contribute to or have the potential to worsen ICH. The first paper reports a randomised-controlled trial of platelet infusion versus the standard care in patients with ICH on antiplatelet agents. The second paper is a randomised-controlled trial of fresh frozen plasma versus prothrombin complex concentrate in the reversal of warfarin-associated ICH. Finally, we review an observational study of ICH associated with NOACs, focussing on prognostic factors and effectiveness of haemostatic treatments.

## Platelet transfusion versus standard care after acute stroke due to spontaneous cerebral haemorrhage associated with antiplatelet therapy (PATCH): a randomised, open-label, phase 3 trial

Observational studies have suggested that antiplatelet drugs contribute to early haematoma expansion and death in the setting of ICH. In addition, there is an independent association between antiplatelet drugs and death from ICH. This randomised, controlled, multicentre trial aimed to compare the addition of platelet transfusion to the standard care.

This European trial included adults on aspirin, clopidogrel, dipyridamole, and carbasalate. ICH was confirmed on first available imaging modality—CT or MRI. Platelets were delivered within 6 h of stroke onset, and 90 min of imaging. Patients with poor premorbid function, suspected focal vascular lesions, extradural or subdural haemorrhages, planned neurosurgical intervention, intraventricular extension, concurrent anticoagulants, infratentorial location, or coma (GCS <8) were excluded. Randomisation was by coin toss; patients were given 1 unit of platelets or 2 if the patient was taking ADP inhibitors. NIHSS was used to grade stroke severity and modified Rankin Scoring (mRS, blinded to treatment allocation) to score outcome. Patents had a 24 h follow-up scan—haemorrhage volume was calculated and checked by blinded raters. Primary outcome was the mRS category change, with secondary outcomes of poor outcome (mRS 3–6), ICH volume, complications of treatment, or complications of the acute event. Data were analysed in an intention to treat basis. An ordinal linear regression analysis was used, changed from a binary outcome analysis prior to statistical analysis [poor outcome (mRS 4–6) versus non-poor outcome] to increase power.

190 participants were included—97 received platelets and 93, standard care. There were no baseline differences between the groups. 42 were excluded (32 for intraventricular haemorrhage, 7 for insufficient imaging, 2 for infratentorial location, and 1 was not taking antiplatelets agents). The primary outcome showed a significant worsening with platelets (OR 1.84, 95 % CI 1.10–3.08; *p* = 0.0200). Serious adverse events were more frequent in the group receiving platelets. No changes were seen on any of the pre-specified subgroup analyses (country, haematoma volume, type of antiplatelet agent).

*Comment*. The sample size for the study was relatively small, with a comparatively high percentage of exclusions; nevertheless, the result seems consistent regardless of the analysis method used, as the secondary outcome also proved significant. The rationale for excluding patients potentially undergoing neurosurgical intervention was not clear and adherence to antiplatelet agents was not measured. Despite these caveats, it is clear that platelet transfusion should be abandoned.

Baharoglu MI et al. (2016) Lancet (Epub ahead of print).

## Fresh frozen plasma versus prothrombin complex concentrate in patients with intracranial haemorrhage related to vitamin K antagonists (INCH): a randomised trial

ICH secondary to warfarin was historically reversed using fresh frozen plasma (FFP), but its status as a blood product limited use in some patient groups and carried associated risks of transfusion reactions and infection. PCC has been shown to be superior owing to faster restoration of normal INR. The two treatments have never been compared in a head to head trial in the setting of ICH.

This randomised-controlled trial recruited adults with an INR of 2.0 or greater diagnosed with a new ICH (within 12 h of neurological symptoms). Patients with a known/suspected vascular malformation, contraindications to large volume transfusion (for example cardiac failure), or poor clinical status (premorbid mRS >2 or GCs <6) were excluded. Data analysis was blinded, as treatment administration could not be. Randomisation was by computer. All participants were given 10 mg of vitamin K, and the trial intervention was delivered within 1 h of diagnostic CT. Haematoma volume was assessed at 3, 24, and 72 h post treatment. The primary endpoint was INR<1.3 at 3 h post infusion. Secondary endpoints were clinical (death, haematoma expansion, NIHSS at discharge, and time to target INR) and functional (EQ5D, mRS, Barthel index, Glasgow outcome scale). Groups were compared using the Chi-squared test.

Twenty-six participants were given FFP and 28 PCC; four had withdrawn (no differences were seen on a sensitivity analysis). The legal authority demanded termination based on an interim analysis showing a worse haematoma expansion in the FFP group. No differences in mortality were seen. Forty-three serious adverse events were reported, although no significant differences were seen between the groups. In a post-hoc analysis, 65 % of the PCC group achieved target INR, whilst none of the FFP group had.

*Comment.* This trial, although stopped early and containing small numbers, is highly suggestive of PCC’s superiority over FFP. However, it may be considered unfortunate that clinical endpoints (death and disability) were not primary endpoints in the trial and that the trial was stopped before it could be determined if any significant differences in these outcomes could be determined.

Steiner T et al. (2016) Lancet Neurol 15:566–573.

## Early clinical and radiological course, management, and outcome of intracerebral haemorrhage related to new oral anticoagulants

Despite the apparent lower frequency of haemorrhage compared with the coumarins, a major limitation of NOACs is the lack of specific reversal therapy if haemorrhage does occur. Our knowledge of the severity and frequency of complications in ‘real world’ settings as opposed to clinical trials is also limited at present. This prospective cohort study aimed to address these gaps.

Participants were recruited from 38 German hospitals between February 1, 2012 and December 31, 2014. Participants were included if they were aged 18 or over, were taking an NOAC, and had ICH at baseline. Investigations and management were left to the treating physician. Clinical data included the NIHSS (National Institute of Health Stroke Score) at 24, 48, and 72 h, mRS (premorbid score, at admission, at discharge and at 90 day follow-up) and the CHA_2_DS_2_-VASC and HASBLED scores. Radiological variables were also included—haematoma expansion (>33 % increase in volume, or absolute increase of 6 ml), which required follow-up imaging within 3–72 h. Groups were compared using Chi-squared tests, Fisher exact test, Mann–Whitney *U* test, and associations were sought using logistic regression.

Sixty-one patients were enrolled from 21 centres. Mena age was 76.1, and mean NIHSS was 10. Median haematoma volume at baseline was 10.8 ml. NIHSS score correlated with haematoma volume. Symptom onset to time of imaging was not correlated with haematoma volume. Haematoma expansion was seen in 38 % of those who had follow-up imaging (45 of the 61 participants), and intraventricular expansion occurred in 11 %.

Thirty-seven of 61 patients received PCC. This had no effect on early haematoma expansion and no difference in mRS at 90 days, although the intervention group had worse clinical status and more frequent deep haemorrhages. Sixteen per cent died as an inpatient, with a 28 % mortality rate at 3 months. 65 % of survivors had poor outcome (mRS 3–5). Strong associations were found between NIHSS score at onset and mRS, as well as baseline haematoma size and mRS.

*Comments.* It is perhaps unsurprising but never-the-less disheartening to see a lack of effect for PCC in this setting. However, it should be noted that these are observational data, the group receiving reversal therapy were more unwell, and the numbers were relatively small. A randomised-controlled trial would answer this question more definitively.

Purrucker JC et al. (2016) JAMA Neurol 73(2):169–177.

